# Intraoperative Intravenous Methadone Versus Non-Methadone Opioid Analgesia in Instrumented Spinal Fusion: A GRADE-Guided Systematic Review and Meta-Analysis

**DOI:** 10.3390/jcm15218470

**Published:** 2026-10-30

**Authors:** Ahmed Abu-Zaid, Reem B. Alhajri, Ghalyah H. Alsuhail, Faisal R. Alabkal, Fatmah Y. Boshehri, Mohammad A. Alshammari, Naser A. Alajmi, Barrak A. Albarrak, Osamah F. Alzufairi, Abdullah F. Alenezi, Yousif T. Alhuwaimel, Abdullah Kh Alabdullah, Abdullah M. Alharran

**Affiliations:** 1 Department of Biochemistry and Molecular Medicine, College of Medicine, Alfaisal University, Riyadh 11533, Saudi Arabia; 2 Faculty of Medicine, Kuwait University, Jabriya 13110, Kuwait; 3 College of Medicine and Medical Sciences, Arabian Gulf University, Manama 22979, Bahrain; 4 Royal College of Surgeons in Ireland, Medical University of Bahrain, Busaiteen 228, Bahrain; 5 Kuwait Institute for Medical Specialization, Kuwait City 13018, Kuwait; 6 School of Medicine, The University of Jordan, Amman 11942, Jordan; 7 GB—School of Medicine, Dentistry and Nursing, University of Glasgow, Glasgow G31 2ER, Scotland, UK; 8 Kuwait National Guard, Medical Services Directorate, Kuwait City 12029, Kuwait

**Keywords:** methadone, spinal fusion, postoperative pain, opioid consumption, opioid-sparing analgesia, perioperative analgesia

## Abstract

**Background:** Instrumented spinal fusion is among the most painful surgical procedures. Methadone, a long-acting μ-opioid receptor agonist with N-methyl-D-aspartate receptor antagonism, may provide more sustained analgesia than shorter-acting intraoperative opioids. **Methods:** PubMed, Scopus, Web of Science, and the Cochrane Library were searched through August 2026 for randomized trials in adults or adolescents undergoing instrumented spinal fusion that compared intraoperative intravenous methadone with a shorter-acting intraoperative opioid. Random-effects meta-analysis, the Cochrane Risk of Bias 2 tool, GRADE, and published minimal clinically important differences (MCIDs) were used. **Results:** Five trials (364 participants; three in adults and two in adolescents) were included. Methadone reduced pain intensity at 24 h (MD −1.06, 95% CI −2.03 to −0.09; three trials), 48 h (MD −1.52, 95% CI −2.36 to −0.67), and 72 h (MD −1.04, 95% CI −1.91 to −0.17; two adult trials each); point estimates approximated the 1-point MCID, but confidence intervals included clinically unimportant differences. Opioid consumption was lower at 24 h (SMD −0.50, 95% CI −0.79 to −0.21; four trials; I^2^ = 0%), corresponding to relative reductions of 17% to 52% in the individual trials, but not at 48 h. Vomiting did not differ (RR 1.10, 95% CI 0.72 to 1.70). Respiratory events were rare, inconsistently defined, and not pooled. Certainty was moderate for 24-h opioid consumption and low or very low for all other outcomes. **Conclusions:** In adults undergoing instrumented spinal fusion, a single intraoperative dose of intravenous methadone may modestly reduce postoperative pain at 24 h and opioid consumption, but the clinical importance of these effects is uncertain; the evidence in adolescents is limited to two small trials, and adverse events were insufficiently reported. Methadone should be regarded as an optional, monitored component of multimodal analgesia rather than routine practice until larger trials powered for safety and longer-term opioid use are available.

## 1. Introduction

Instrumented spinal fusion is performed to restore spinal stability and correct deformity, but it is associated with severe postoperative pain because of extensive muscle dissection, bone work, and instrumentation [1]. Patients undergoing major spinal procedures frequently report moderate-to-severe pain during the early postoperative period, making effective analgesia an important component of recovery [2]. Inadequately controlled pain may delay mobilization, prolong hospitalization, and reduce satisfaction, and it is associated with persistent postsurgical pain [3].

Opioids remain central to analgesia after spinal fusion, particularly during the first postoperative days [4]. However, commonly used short- and intermediate-acting opioids may require repeated administration or patient-controlled analgesia to maintain pain relief. Increasing opioid exposure may also produce nausea, vomiting, pruritus, sedation, respiratory depression, ileus, and delayed recovery [5]. These concerns have encouraged multimodal and opioid-sparing strategies that provide sustained analgesia without increasing perioperative complications [6].

Methadone is a long-acting μ-opioid receptor agonist with properties that may be useful in instrumented spinal fusion [4]. In addition to its prolonged duration of action, methadone antagonizes N-methyl-D-aspartate receptors and inhibits serotonin and norepinephrine reuptake, mechanisms that may reduce sensitization, tolerance and opioid-induced hyperalgesia [7]. A single intravenous dose administered intraoperatively may therefore provide analgesia that extends beyond the recovery room and reduce the need for supplemental postoperative opioids.

Randomized trials of intraoperative methadone in spinal fusion have, however, produced inconsistent findings, ranging from lower pain scores and opioid requirements to opioid sparing without better pain control, or no measurable benefit [4,5,6,8,9]; the individual trials are considered in the Results and Discussion. We focused on instrumented spinal fusion because it combines extensive muscle dissection, osteotomy, and instrumentation with intense and prolonged nociceptive input [1,2], so that it is the procedure in which a long-acting intraoperative opioid is most plausibly useful, and because its analgesic context differs from that of other procedures, including long-standing concerns about the use of nonsteroidal anti-inflammatory drugs after instrumented fusion [10].

Interpretation of this evidence is limited by small sample sizes, differences between adult and adolescent populations, variation in methadone dose and timing, heterogeneous comparator opioids, and inconsistent definitions of opioid consumption and pain outcomes. Previous meta-analyses pooled methadone trials from cardiac, abdominal, orthopedic, and spinal procedures [11,12], the available syntheses that focus on orthopedic or spine surgery are scoping reviews without quantitative pooling [13], and the two most recent spinal fusion trials, including the largest to date, were not included in any of them [5,8]. A focused synthesis of randomized evidence is therefore needed to clarify whether intraoperative intravenous methadone offers clinically meaningful benefits over shorter-acting opioid analgesia in instrumented spinal fusion. This systematic review and meta-analysis aimed to compare the effects of intraoperative intravenous methadone with non-methadone opioid regimens on postoperative opioid consumption, pain intensity, and opioid-related adverse events in patients undergoing instrumented spinal fusion.

## 2. Methods

### 2.1. Study Design and Reporting

This systematic review and meta-analysis assessed randomized trials of intraoperative intravenous methadone in patients undergoing instrumented spinal fusion. Reporting followed the Preferred Reporting Items for Systematic Reviews and Meta-Analyses framework and the Cochrane guidance for intervention reviews [14,15]. The review protocol was registered prospectively in PROSPERO [CRD420261494657].

### 2.2. Search Strategy

The electronic search covered PubMed, Scopus, Web of Science, and the Cochrane Library from inception through 27 August 2026. Search terms combined methadone and its synonyms with terminology for spinal fusion, including posterior, thoracolumbar, and lumbar fusion, spondylodesis, and spondylosyndesis. Medical Subject Headings were incorporated when available. Full database-specific strategies and retrieval counts are provided in Supplementary Table S1.

### 2.3. Eligibility Criteria

We included randomized controlled trials involving adults or adolescents undergoing instrumented spinal fusion, provided that intravenous methadone was administered during surgery, that the comparator was a non-methadone intraoperative opioid regimen, and that at least one eligible postoperative outcome was reported. Trials in which methadone was given to all arms, including trials that tested an adjunct such as ketamine on a background of methadone, were ineligible because they do not address the methadone versus non-methadone question.

The comparator was defined pragmatically as the shorter-acting opioid (sufentanil, remifentanil, hydromorphone, or morphine) that each trial used as its standard intraoperative analgesic. These agents differ in potency and pharmacokinetics, but all are conventional μ-opioid receptor agonists that lack the prolonged elimination half-life and N-methyl-D-aspartate receptor antagonism that distinguish methadone, and each was dosed according to the usual practice of the trial. The review question was therefore whether substituting methadone for the usual shorter-acting intraoperative opioid improves outcomes, which is the decision clinicians face and the approach taken by previous meta-analyses of intraoperative methadone [11,12]. The comparator used in each trial is reported in Table 1 and in the forest plots, and the consistency of effects across comparators was examined descriptively (Section 2.7).

Outcomes of interest were postoperative opioid use, pain severity, vomiting, respiratory events, sedation, hypotension, arrhythmia, nausea, and hospital stay. We excluded nonrandomized research, studies enrolling an unsuitable surgical population, conference abstracts lacking adequate outcome information, and reports without a relevant comparative arm.

### 2.4. Study Selection and Data Extraction

After EndNote-based duplicate removal [16], two reviewers independently screened titles and abstracts and then evaluated the full texts of potentially relevant reports against the eligibility criteria. Screening was performed in Rayyan [17]. Any disagreement was resolved through consultation with a third reviewer.

Data collection covered the publication year, country, trial design, registration information, study and follow-up duration, eligibility criteria, participant profile, sample size, operative population, study treatments, comparator regimen, methadone dose and timing, and reported postoperative outcomes. Baseline information included age, sex, body mass index, weight, height, and American Society of Anesthesiologists physical status.

For trials with more than two arms, pairwise comparisons were selected to avoid counting a comparator group twice. The methadone-plus-remifentanil arm was paired with remifentanil alone in Martin et al., whereas the pre-incisional methadone arm was paired with morphine in Uhrbrand et al. Continuous variables were recorded as means with standard deviations.

### 2.5. Risk of Bias Assessment

The Cochrane Risk of Bias 2 tool was used to judge study quality [18]. The assessment covered randomization, departures from the assigned intervention, missing outcome data, outcome measurement, and selective reporting. Each domain was rated as low risk, some concerns, or high risk.

### 2.6. Outcomes

The prespecified primary outcomes were postoperative opioid consumption and pain intensity at 24, 48, and 72 h. Pain was assessed with a visual analog or numerical rating scale from 0 to 10, where higher values represented more severe pain. Vomiting and respiratory events were the safety outcomes. Respiratory events were extracted as defined by each trial (for example, respiratory depression or hypoventilation); because the definitions and observation windows differed between trials, these events were synthesized at the trial level without pooling (Section 2.7). Other adverse events, including nausea, sedation, pruritus, constipation, dizziness, and cardiac events, were summarized narratively because they were reported inconsistently. Opioid consumption and pain after hospital discharge were recorded descriptively when reported.

### 2.7. Statistical Analysis

Pain data reported on comparable 0-to-10 scales were combined as mean differences (MD) with 95% confidence intervals. Postoperative opioid consumption was reported in different opioids and units (milligrams of intravenous or oral morphine equivalents, or milligrams per kilogram of hydromorphone or morphine equivalents) and with different methods of normalization. Rather than converting these values with equianalgesic tables, which are themselves uncertain, each trial’s effect was expressed as a standardized mean difference (SMD), which divides the between-group difference by the pooled standard deviation and thereby places all trials on a common, unit-free scale [15,19]. SMDs of approximately 0.2, 0.5, and 0.8 are conventionally regarded as small, moderate, and large effects [19]. To aid clinical interpretation, the relative reduction in mean opioid consumption observed in each trial is also reported. Risk ratios (RR) with 95% confidence intervals were calculated for vomiting. Respiratory events were not pooled because the two reporting trials used different definitions and observation windows; their results are presented separately.

Random-effects inverse-variance models were selected in anticipation of differences in participants, operations, methadone regimens, and comparator opioids. A negative mean or standardized mean difference favored methadone, as did a risk ratio below 1 for adverse events. Heterogeneity was examined with I^2^ and tau2. Statistical significance was defined by a two-sided *p*-value below 0.05.

Subgroup analyses by age group (adults versus adolescents) and by comparator opioid were considered but not performed, because no outcome was informed by more than four trials and most subgroups would have contained a single trial. Subgroup comparisons based on so few studies have negligible power and a high risk of spurious findings, and they are discouraged by the Cochrane Handbook [15]. Instead, the consistency of effects across adult and adolescent trials and across comparators was examined descriptively, using the trial-level estimates, the heterogeneity statistics, and the leave-one-out analyses, in which omission of an adolescent trial yields the pooled estimate for the remaining adult trials.

Leave-one-out analyses were performed for 24-h pain and opioid consumption. Small-study effects were explored with Doi plots and the Luis Furuya-Kanamori (LFK) index [20,21]. The Doi plot is a folded normal quantile plot in which each study’s effect estimate is plotted against the absolute value of a normal quantile (Z-score) derived from its precision-weighted rank; in the absence of bias, the studies form a symmetrical, mountain-shaped curve with the most central estimate at the apex, whereas small-study effects skew the curve to one side. The LFK index quantifies this deviation from symmetry: values within ±1 indicate no asymmetry, values beyond ±1 but within ±2 minor asymmetry, and values beyond ±2 major asymmetry [21]. The method was chosen instead of a funnel plot and Egger’s test because it retains some discriminatory ability with few studies [20,21], although its properties have been questioned [22] and, with fewer than five studies, any assessment of small-study effects is exploratory. Analyses were conducted in R with the meta and dmetar packages (R Foundation for Statistical Computing, Vienna, Austria) [23,24,25].

### 2.8. GRADE Certainty of Evidence

Certainty for each primary outcome was judged with the GRADE approach [26]. Randomized evidence began at high certainty and was downgraded when concerns involved risk of bias, inconsistency, indirectness, imprecision, or publication bias. Ratings were classified as high, moderate, low, or very low. Imprecision was judged against the minimal clinically important difference (MCID): for pain intensity, a difference of 1 point on a 0-to-10 scale, corresponding to the 10-mm threshold derived for acute postoperative pain [27] and to the lower end of the thresholds used in trials of postoperative analgesia [28]; for opioid consumption, the thresholds most commonly used in such trials, namely an absolute reduction of 10 mg of intravenous morphine equivalents or a relative reduction of 30% to 40% over 24 h. Because opioid consumption was pooled as an SMD, the relative reductions observed in the individual trials were used for this judgement. Publication bias was considered exploratory because fewer than 10 studies contributed to each outcome, so no downgrade was applied for that domain.

## 3. Results

### 3.1. Study Selection

**Figure 1:**
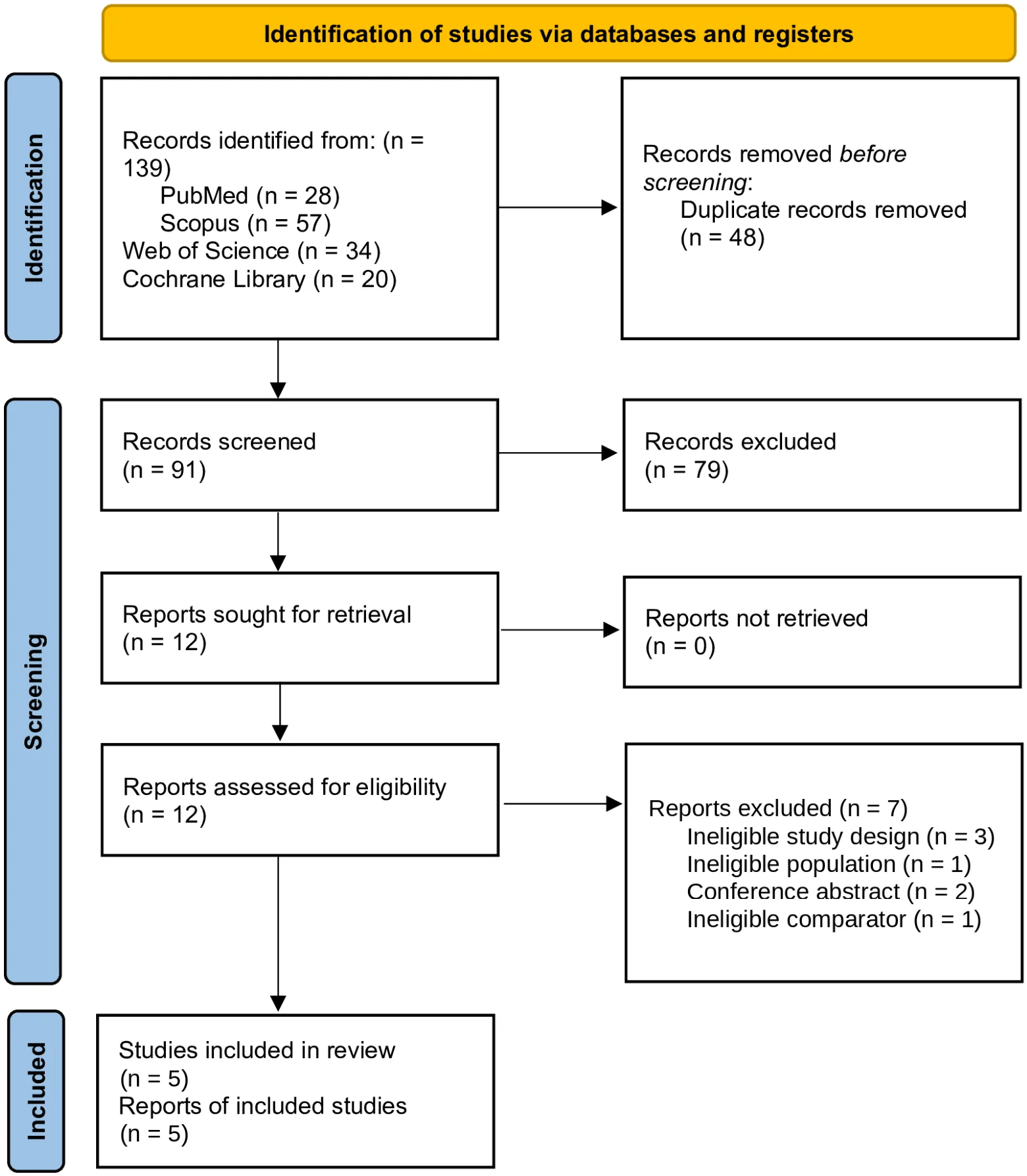
PRISMA 2020 flow diagram of study selection.

The search yielded 139 records, including 28 from PubMed, 57 from Scopus, 34 from Web of Science, and 20 from the Cochrane Library. Removal of 48 duplicates left 91 records for title and abstract screening. After 79 records were excluded, 12 reports underwent full-text review, all of which were retrieved. Seven reports were subsequently excluded because of ineligible design (*n* = 3), ineligible population (*n* = 1), conference-abstract status (*n* = 2), or an ineligible comparator (*n* = 1; a trial in which both arms received methadone and ketamine was added to one arm [29]). Five randomized trials entered the systematic review [4,5,6,8,9]. The selection pathway is presented in Figure 1.

### 3.2. Study Characteristics

The five eligible trials were published from 2011 to 2025 and provided data for 364 analyzed participants. Trial sizes ranged from 29 to 124 randomized participants. Three trials recruited adults undergoing elective multilevel thoracolumbar [4], posterior lumbar or thoracic [9], or lumbar [8] instrumented fusion, whereas two enrolled adolescents undergoing posterior fusion for idiopathic scoliosis [5,6]. The indications for surgery therefore differed between the adult trials (predominantly degenerative lumbar disease in Uhrbrand et al., who excluded fusion for malignancy or over more than four levels) and the adolescent trials (deformity correction), and they were not reported in a uniform way. Preoperative pain and opioid exposure, which influence postoperative pain, were also inconsistently reported: Uhrbrand et al. excluded patients using more than 60 mg of oral morphine equivalents daily [8], Murphy et al. and Fons et al. excluded patients with a recent history of opioid or substance misuse [5,9], and preoperative pain intensity was not consistently reported as a baseline characteristic.

Intravenous methadone doses ranged from 0.1 to 0.2 mg/kg. In most trials it was given once during surgery; Fons et al. divided 0.2 mg/kg between administration before incision and before wound closure [5]. Comparator opioids were sufentanil [4], hydromorphone [9], remifentanil [6], and morphine [5,8], each given according to the standard intraoperative practice of the trial. Background analgesia also differed: in the two earlier adult trials, postoperative analgesia relied on opioid patient-controlled analgesia without scheduled paracetamol or nonsteroidal anti-inflammatory drugs [4,9], whereas the more recent trials embedded methadone in multimodal regimens that included scheduled non-opioid analgesics [5,8]. In-hospital follow-up of the pooled outcomes extended to 72 postoperative hours in all trials. Uhrbrand et al. also followed patients for three months and found no between-group differences in opioid requirements or pain at that time [8], and the one-year follow-up of the trial by Murphy et al., reported separately, found a lower weekly frequency of persistent pain with methadone at three months but not at six or twelve months [30]. Study and baseline details are summarized in Table 1 and Table 2.

### 3.3. Risk of Bias

**Figure 2:**
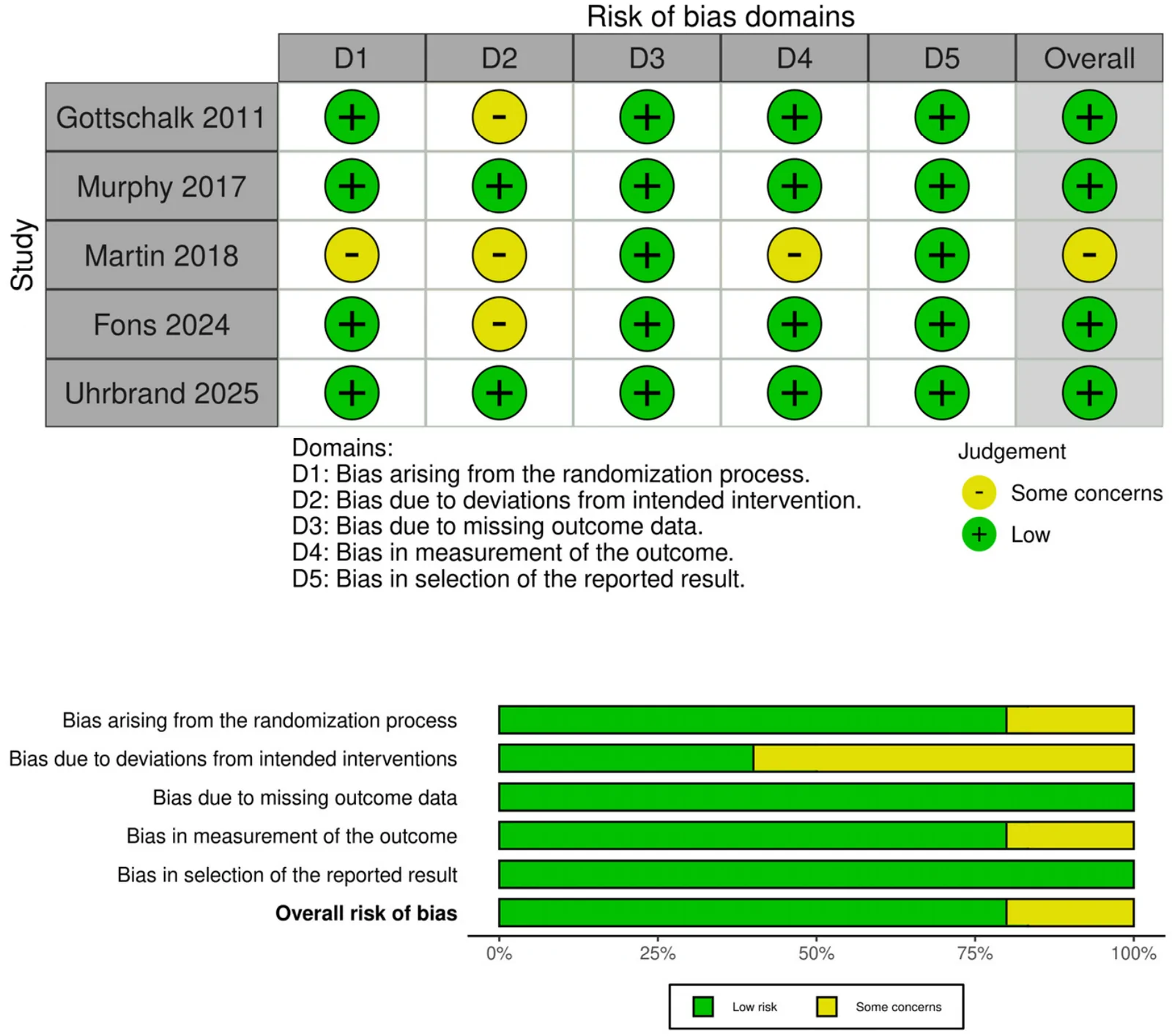
Risk-of-bias assessment of the included RCTs [4,5,6,8,9].

Two trials, Murphy et al. and Uhrbrand et al., were judged to have low risk of bias in all five domains [8,9]. Some concerns about deviations from the intended intervention were identified in Gottschalk et al. and Fons et al. Martin et al. raised concerns involving randomization, deviations from the assigned intervention, and outcome measurement. No study received a high-risk judgment in any domain. Study-level and domain-level judgments are shown in Figure 2.

### 3.4. Main Analysis

#### 3.4.1. Postoperative Pain Intensity

**Figure 3:**
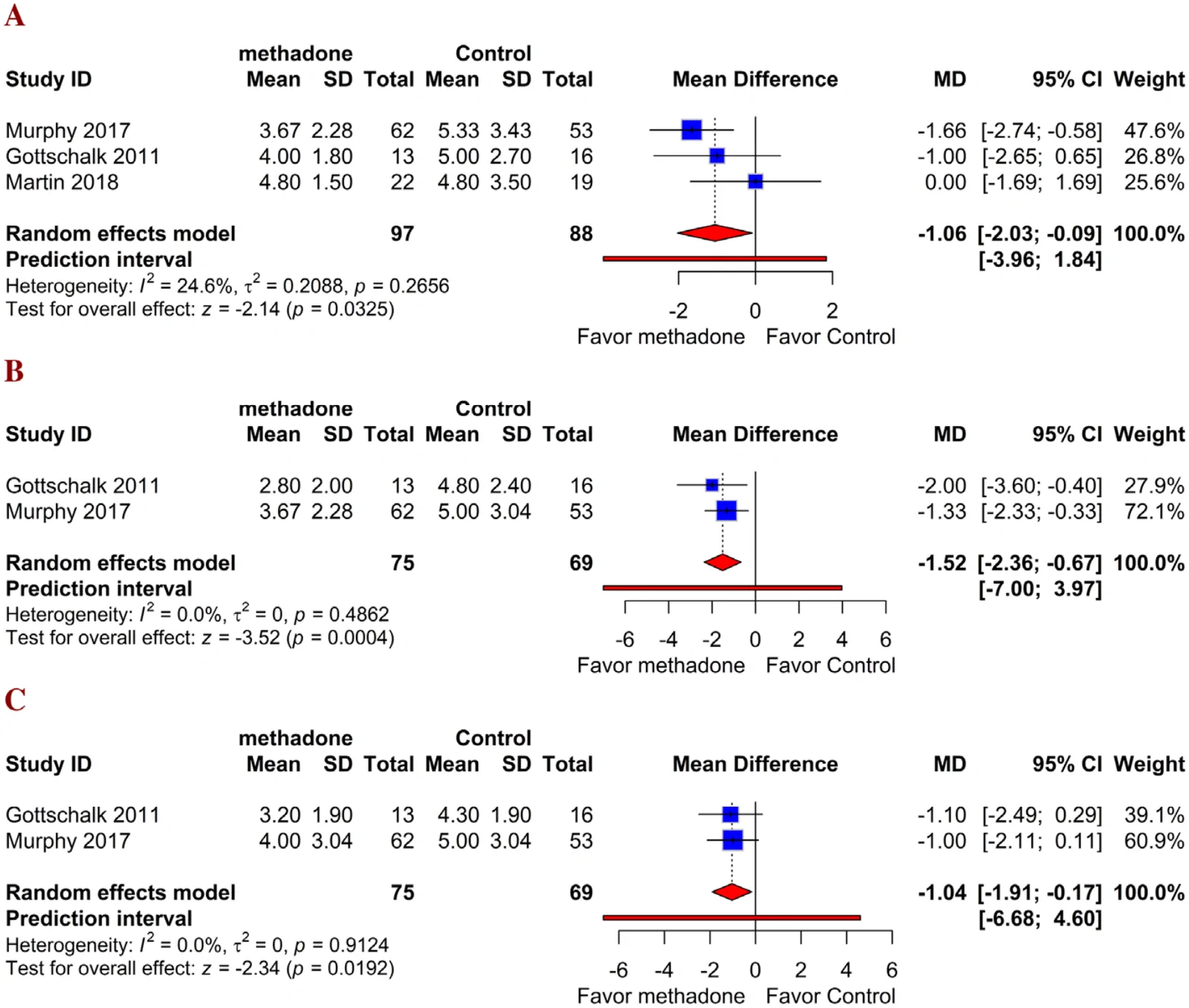
Forest plots of postoperative pain intensity: (**A**): Pain intensity at 24 h, (**B**): Pain intensity at 48 h, (**C**): Pain intensity at 72 h [4,6,9].

Three trials involving 185 participants, two in adults and one in adolescents, supplied data for pain intensity at 24 h. Compared with non-methadone opioid analgesia, intraoperative methadone produced lower pain scores (MD, −1.06; 95% CI, −2.03 to −0.09; *p* = 0.033), with low heterogeneity (I^2^ = 24.6%; Figure 3A).

At 48 h, two adult trials involving 144 participants showed lower pain intensity with methadone (MD, −1.52; 95% CI, −2.36 to −0.67; *p* < 0.001). No statistical heterogeneity was detected (I^2^ = 0%; Figure 3B).

The same two adult trials contributed data at 72 h. Pain remained lower after methadone (MD, −1.04; 95% CI, −1.91 to −0.17; *p* = 0.019), with I^2^ = 0% (Figure 3C). However, the limited number of studies produced wide prediction intervals for both the 48- and 72-h estimates. At all three time points, the point estimates were close to or slightly larger than the 1-point MCID, whereas the confidence intervals included differences smaller than 1 point (Section 3.4.6).

#### 3.4.2. Postoperative Opioid Consumption

**Figure 4:**
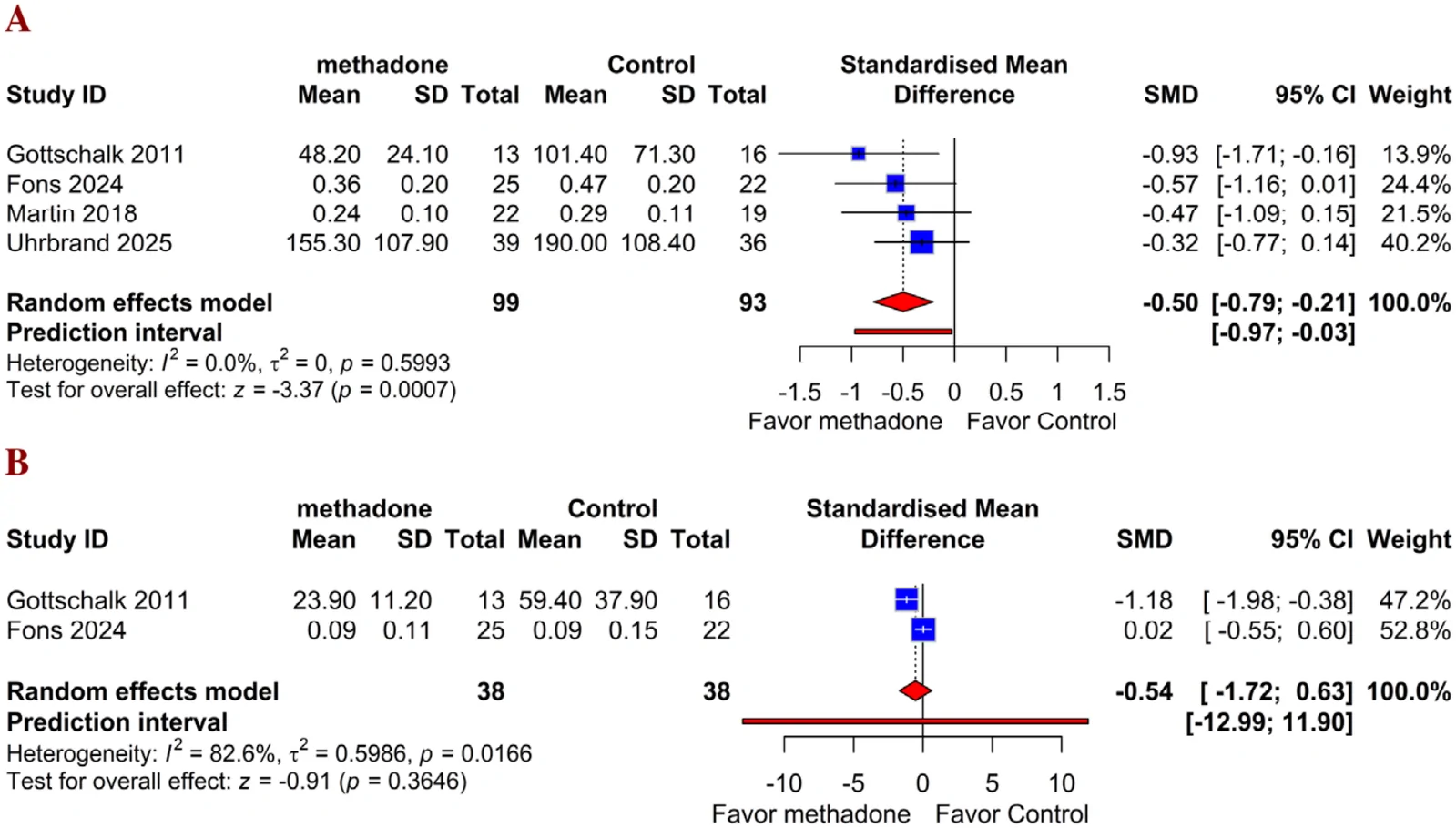
Forest plots of postoperative opioid consumption: (**A**): Opioid consumption at 24 h, (**B**): Opioid consumption at 48 h [4,5,6,8].

Four trials with 192 participants, two in adults and two in adolescents, contributed to the 24-h opioid analysis. Intraoperative methadone was associated with lower postoperative opioid consumption than non-methadone regimens (SMD, −0.50; 95% CI, −0.79 to −0.21; *p* < 0.001), without observed heterogeneity (I^2^ = 0%; Figure 4A). Relative to the comparator group, mean 24-h opioid consumption in the methadone group was lower by 52% in Gottschalk et al. (48.2 versus 101.4 mg of morphine equivalents), 23% in Fons et al. (0.36 versus 0.47 mg/kg), 17% in Martin et al. (0.24 versus 0.29 mg/kg of hydromorphone), and 18% in Uhrbrand et al. (155.3 versus 190.0 mg of oral morphine equivalents).

Two trials involving 76 participants, one in adults and one in adolescents, contributed at 48 h. The pooled comparison did not show a statistically significant difference in opioid use (SMD, −0.54; 95% CI, −1.72 to 0.63; *p* = 0.365), and heterogeneity was substantial (I^2^ = 82.6%; Figure 4B).

#### 3.4.3. Adverse Events

**Figure 5:**
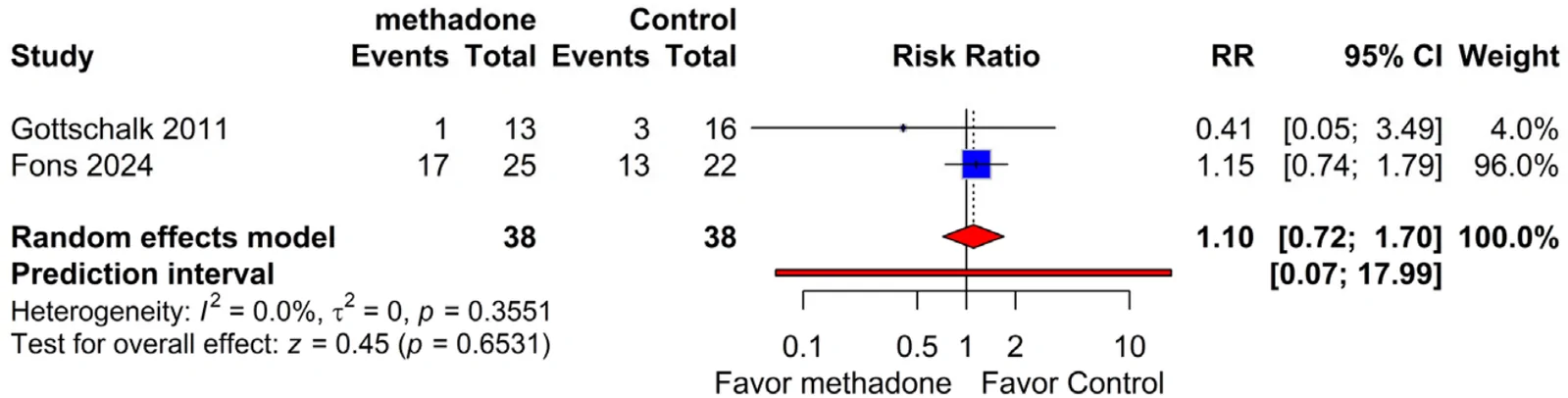
Forest plot of postoperative vomiting [4,5].

Vomiting was reported in two trials. The risk did not differ between methadone and non-methadone opioid analgesia (RR, 1.10; 95% CI, 0.72 to 1.70; *p* = 0.653; I^2^ = 0%; Figure 5).

Respiratory events were reported by two trials with different definitions and observation windows and were therefore not pooled. Uhrbrand et al. recorded hypoventilation (respiratory rate below 10 breaths per minute) in the post-anesthesia care unit as a prespecified adverse event; in the extracted data, events occurred in 2 of 39 participants given pre-incisional methadone and 5 of 14 participants given morphine (RR, 0.14; 95% CI, 0.03 to 0.66), although the trial authors described adverse events overall as rare and not different between groups [8]. Gottschalk et al. reported respiratory depression during the 72-h postoperative period in none of 13 methadone-treated and 1 of 16 sufentanil-treated participants (RR, 0.41; 95% CI, 0.02 to 9.21) [4]. With eight events in total, these data cannot establish whether methadone reduces or increases the risk of respiratory events.

Other opioid-related adverse events were reported heterogeneously. Where trial authors summarized opioid-related side effects, they described their incidence as comparable between groups [4,5,8]; however, sedation, dizziness, constipation, and cardiac safety outcomes such as QT-interval prolongation or arrhythmia were not reported in a form that allowed extraction or pooling, and no trial was powered for adverse events.

#### 3.4.4. Consistency of Effects Across Adult and Adolescent Trials

Because formal subgroup analyses were not feasible (Section 2.7), the contribution of adult and adolescent trials to each outcome was examined descriptively. The 48-h and 72-h pain analyses included adult trials only [4,9]. The 24-h pain analysis included one adolescent trial, Martin et al., which found no difference in pain scores (MD, 0.00; 95% CI, −1.69 to 1.69) [6]; when this trial was omitted, the estimate for the two adult trials was MD −1.46 (95% CI, −2.37 to −0.56; I^2^ = 0%; Supplementary Figure S1). In the 24-h opioid-consumption analysis, the estimates of the two adolescent trials (SMD, −0.57 and −0.47) [5,6] lay within the range of the two adult trials (SMD, −0.93 and −0.32) [4,8], heterogeneity was absent (I^2^ = 0%), and the pooled effect persisted when either adolescent trial was omitted (Supplementary Figure S2). The 48-h opioid analysis comprised one adult and one adolescent trial whose estimates diverged (SMD, −1.18 versus 0.02); because these trials also differed in comparator, methadone regimen, and background analgesia, this heterogeneity cannot be attributed to age. The 24-h opioid-sparing effect was likewise consistent in direction across the four comparator opioids (Figure 4A). These descriptive comparisons do not replace adequately powered subgroup analyses, and age-specific effects remain uncertain.

#### 3.4.5. Sensitivity and Small-Study-Effect Analyses

The 24-h pain estimate depended on the two adult trials, which together carried 74% of the weight. Statistical significance was lost when either Murphy et al. (MD, −0.51; 95% CI, −1.69 to 0.67) or Gottschalk et al. (MD, −0.96; 95% CI, −2.57 to 0.64) was omitted, whereas omission of the adolescent trial by Martin et al., whose null result carried the least weight, increased the pooled effect (MD, −1.46; 95% CI, −2.37 to −0.56; Supplementary Figure S1).

The 24-h opioid-consumption finding was more stable. Its direction and statistical significance were retained after removal of each study. Leave-one-out SMDs ranged from −0.43 to −0.62, and I^2^ remained 0% in every iteration (Supplementary Figure S2).

The Doi plot for 24-h pain had an LFK index of 1.16 and the plot for 24-h opioid consumption an index of −1.81, both within the range conventionally interpreted as minor asymmetry (Supplementary Figures S3 and S4). In the pain plot, the asymmetry reflected the least precise trial, Martin et al., which reported a null effect; in the opioid-consumption plot, the most precise trial, Uhrbrand et al., reported the smallest effect and the least precise trial, Gottschalk et al., the largest, a pattern that is compatible with small-study effects but equally with genuine differences between trials in design and background analgesia. With only three and four studies, these plots cannot distinguish publication bias from chance or clinical heterogeneity, and they were not used to downgrade the certainty of evidence.

#### 3.4.6. GRADE Assessment

GRADE certainty was moderate for 24-h opioid consumption and was downgraded for risk of bias. Certainty for pain intensity at 24, 48, and 72 h was low because of risk-of-bias concerns and imprecision; the confidence intervals included differences smaller than the 1-point MCID. Vomiting was also supported by low-certainty evidence for the same reasons. Certainty for 48-h opioid consumption was very low because of risk of bias, marked heterogeneity (I^2^ = 82.6%), and imprecision. The trial-level evidence on respiratory events was rated very low because of risk of bias, differences in outcome definitions and follow-up, and very serious imprecision caused by few events (Supplementary Table S2).

## 4. Discussion

In this meta-analysis of five randomized trials, intraoperative administration of intravenous methadone at doses of 0.1–0.2 mg/kg was associated with lower pain scores at 24, 48, and 72 h and with lower opioid consumption during the first 24 h after instrumented spinal fusion, compared with shorter-acting intraoperative opioids. These effects were modest. The pooled reductions in pain intensity (1.0 to 1.5 points on a 0-to-10 scale) were close to the MCID of approximately 1 point reported for acute postoperative pain [27,28], but their confidence intervals included differences well below that threshold, and the two later time points were informed by only two adult trials. The opioid-sparing effect at 24 h (SMD, −0.50) corresponded to relative reductions of 17% to 52% in the individual trials; only the smallest and earliest trial exceeded the 40% relative reduction that is commonly regarded as clinically important [28], whereas the two most recent trials, including the largest, showed reductions of about 20% [5,8]. No opioid-sparing effect was demonstrable at 48 h. The certainty of evidence was moderate for 24-h opioid consumption and low or very low for all other outcomes. The findings therefore indicate a possible, modest, and mainly early benefit rather than a sustained and clinically established one.

A pharmacological explanation for an early benefit is plausible. Methadone has an elimination half-life of about 24 to 36 h, so that a single intraoperative dose maintains relatively stable plasma concentrations during the period in which postoperative pain is most intense, whereas repeated doses of shorter-acting opioids produce fluctuating concentrations with alternating periods of inadequate analgesia and excessive opioid effect [31,32,33]. Its N-methyl-D-aspartate receptor antagonism may additionally limit central sensitization and opioid-induced hyperalgesia [33]. More stable analgesia, rather than a large absolute reduction in opioid exposure, may therefore be the more relevant mechanism, and it would also be expected to reduce the side effects that accompany peak concentrations after repeated rescue doses, such as sedation and nausea. Pharmacology does not, however, translate directly into clinical outcomes: in the included trials, opioid sparing was not consistently accompanied by lower pain scores [5,6], and lower pain scores were not consistently accompanied by opioid sparing [8]. Postoperative opioid consumption also depends on nursing practice, patient-controlled analgesia settings, background analgesia, and patient expectations, which limits its value as a surrogate for analgesic efficacy.

Beyond the pain score itself, severe pain after spinal surgery interferes with coughing, deep breathing, sleep, mobilization, and neurological assessment [34]. Whether the modest improvements observed here translate into earlier mobilization or better functional recovery was not assessed by the included trials.

The direction of our findings agrees with previous meta-analyses of intraoperative methadone, which pooled cardiac, abdominal, orthopedic, and spinal procedures and reported lower pain scores and opioid consumption during the first 72 h [11,12]. Those reviews, however, included at most three of the five trials analyzed here, namely those published up to 2018 [4,6,9], and the more recent scoping review of orthopedic surgery did not pool data [13]. The present analysis adds the two most recent trials [5,8], including the largest trial to date, which found no reduction in opioid requirements with a single dose of methadone compared with morphine [8]. These two trials contribute almost two-thirds of the participants in the 24-h opioid analysis and are the main reason why our estimates are smaller and more uncertain than those of the earlier reviews.

The spine-specific focus of the present analysis is relevant because the benefit of methadone appears to depend on the intensity and duration of surgical pain. The recent scoping review of perioperative methadone in orthopedic surgery found that the benefits were concentrated in major spine surgery, whereas in two trials of arthroscopic knee surgery, a less painful procedure in which methadone was given intra-articularly, morphine provided superior analgesia; the authors concluded that the analgesic advantage of methadone is less pronounced or absent in lower-pain procedures [13]. Methadone should therefore not be regarded as a universal intraoperative opioid, and even in major spine surgery its use has to be weighed against the modest and uncertain benefit described above.

The opioid-sparing effect should not be overstated. Relative reductions of about 20% in the two most recent trials are smaller than the 30% to 40% reductions that are usually considered clinically important in trials of postoperative analgesia [28], and the absolute differences in the adolescent trials (0.05 to 0.11 mg/kg over 24 h) were small [5,6]. The N-methyl-D-aspartate receptor antagonism of methadone may reduce opioid-induced hyperalgesia and improve the response to subsequent analgesic treatment [35], but any resulting reduction in rescue-opioid exposure is unlikely to be large enough, by itself, to reduce sedation, nausea, constipation, or urinary retention appreciably. The more plausible advantage of a long-acting opioid is the more stable analgesia discussed above, which was not directly tested by any included trial.

The absence of a demonstrable opioid-sparing effect at 48 h does not necessarily contradict the early benefit. After the first postoperative day, opioid requirements are influenced by wound healing, mobilization, preoperative opioid exposure, psychological factors, scheduled non-opioid analgesics, and the transition from intravenous to oral medication [36,37], all of which dilute the contribution of a single intraoperative dose. The 48-h analysis was also based on only two small trials with divergent results. Taken together with the pharmacokinetic profile of methadone, the data suggest that any benefit is concentrated in the first 24 to 48 postoperative hours.

The safety data are the main weakness of the current evidence. Vomiting did not differ between groups in the two trials that reported it, but the confidence interval was wide and the estimate was dominated by one adolescent trial. Respiratory events were reported by only two trials, with different definitions and observation windows, and the eight events observed preclude any conclusion about relative safety; these outcomes were therefore not pooled. Sedation, dizziness, constipation, pruritus, urinary retention, and cardiac events such as QT-interval prolongation and arrhythmia were reported inconsistently or not at all, and no trial was powered for adverse events. Because a long-acting opioid can be expected to reduce the need for shorter-acting opioids, the clinically decisive question is not whether methadone is opioid-sparing but whether it is at least as safe as the regimen it replaces, and this question remains unanswered.

The long and variable elimination half-life of methadone creates a theoretical risk of prolonged sedation and delayed respiratory depression, particularly when it is combined with other central nervous system depressants, and methadone also prolongs the QT interval in a dose-dependent manner [33]. Prescribing information and patient-safety guidance emphasize the need for appropriate dosing and monitoring because respiratory depression may persist beyond, and may not coincide with, the peak analgesic effect [38,39]. Accordingly, the current findings support cautious use under appropriate monitoring, with exclusion of patients at risk of QT prolongation or opioid-induced ventilatory impairment, rather than unrestricted adoption.

### 4.1. Clinical Implications

Current procedure-specific guidance supports multimodal analgesia after complex spine surgery. The PROSPECT recommendations advise paracetamol and NSAIDs or COX-2 selective inhibitors administered before or during surgery and continued postoperatively, an intraoperative low-dose intravenous ketamine infusion, and epidural analgesia with local anesthetics with or without opioids, with opioids reserved for rescue analgesia [40].

The same guidance explicitly does not recommend intravenous methadone, because the supporting trials compared it with shorter-acting opioids, did not use non-opioid background analgesia, and raised safety concerns [40]. Our findings do not contradict this position. Two of the five included trials, including the trial with the largest opioid-sparing effect, did not use scheduled paracetamol or NSAIDs [4,9]; reluctance to prescribe NSAIDs after instrumented fusion reflects long-standing concerns about bone healing, although short perioperative courses appear to be safe [10]. Where methadone was added to multimodal analgesia, its incremental effect was smaller [5,8]. Methadone should therefore not replace multimodal analgesia. At most, it may be considered as one optional component of a monitored multimodal pathway for selected adults in whom severe pain is expected, and its routine use cannot be recommended on current evidence.

The ERAS Society consensus for lumbar spinal fusion likewise emphasizes standardized multimodal analgesia and coordinated perioperative care [41]. The present findings may justify further evaluation of methadone within such pathways, with adequate monitoring, but they do not justify its adoption as a routine component of them.

### 4.2. Strengths, Limitations, and Recommendations

The main strengths of this meta-analysis were the restriction to randomized controlled trials, the spine-specific population, the prospectively registered protocol, and the assessment of pain, opioid consumption and adverse events at defined postoperative time points. The analysis also incorporated risk-of-bias assessment, GRADE evaluation, leave-one-out sensitivity analyses, exploration of small-study effects, and interpretation of effect sizes against MCIDs. Trials in which methadone was given to all arms were excluded so that the comparison addresses a single clinical question.

Several limitations should be considered. The evidence base is small: five trials, none with more than 124 participants and no more than four contributing to any outcome, and two trials had some concerns in one or more risk-of-bias domains. The trials enrolled both adults and adolescents, used four different comparator opioids, different methadone regimens, and different background analgesic regimens, and had different surgical indications; with so few trials, these sources of clinical heterogeneity could only be examined descriptively, and the possibility that the effect of methadone differs between adults and adolescents, or between comparator opioids, cannot be excluded. In particular, the only adolescent trial that contributed to the pain analysis found no benefit, and the 48-h opioid analysis comprised one adult and one adolescent trial with divergent results. Preoperative pain intensity and opioid exposure, which influence postoperative pain, were not consistently reported. The pooled outcomes were limited to the first 72 h, so the effect of methadone on opioid use after discharge, on persistent postsurgical pain, and on longer-term opioid exposure, which are of particular concern in the current opioid epidemic, could not be assessed; only two trials reported longer-term outcomes, with inconsistent results [8,30]. Adverse-event reporting was heterogeneous and underpowered; respiratory events could not be pooled, and sedation, dizziness, constipation, and cardiac safety were not systematically captured. Finally, the small number of trials limits the reliability of the analyses of small-study effects, and the LFK index itself has been criticized in simulation studies [22].

Future trials should be powered for safety and efficacy and should evaluate methadone within clearly defined multimodal and ERAS pathways, using standardized dosing, timing, rescue analgesia, and outcome definitions, including a uniform definition of respiratory depression and systematic recording of sedation, dizziness, constipation, QT-interval prolongation, and arrhythmia. They should report preoperative pain and opioid use, follow patients beyond discharge to capture opioid consumption and persistent pain, and enroll adults and adolescents in sufficient numbers to permit prespecified subgroup analyses by age, comparator opioid, preoperative opioid exposure, and procedure magnitude.

## 5. Conclusions

In adults undergoing instrumented spinal fusion, intraoperative administration of intravenous methadone at doses of 0.1–0.2 mg/kg may modestly reduce pain scores and early opioid consumption compared with shorter-acting opioids, but the size of the benefit is of uncertain clinical importance, the certainty of the evidence is low for most outcomes, the data in adolescents are limited to two small trials, and the safety outcomes that matter most, namely respiratory depression, sedation, and cardiac events, were insufficiently reported. The current evidence does not support routine use. Methadone may be considered as one optional, monitored component of a multimodal analgesic pathway for selected patients in whom severe pain is expected, and adequately powered trials with standardized safety outcomes and follow-up beyond discharge are needed before firmer recommendations can be made.

## Figures and Tables

**Table 1:** Summary characteristics of the included studies.

Study ID	Design	Registration No.	Country	Study Duration	Follow-Up	Inclusion Criteria	Total N	Intervention Group			Control Group		
								Name	Dose	n	Name	Dose	n
Gottschalk 2011 [4]	Randomized controlled trial	NR	Germany	NR	3 days	Patients aged 18–75 undergoing thoracolumbar spine surgery with fusion	29	Methadone	0.2 mg/kg infusion after the sufentanil 0.75 ug/kg induction	13	Sufentanil	0.75 ug/kg before operation then 0.25 ug/kg infusion	16
Murphy 2017 [9]	Randomized controlled trial	NCT02107339	USA	3 March 2014 to 6 June 2016	3 days	Patients aged 18–80 undergoing spine fusion	120	Methadone	0.2 mg/kg infusion	63	Hydromorphone	2 mg	57
Martin 2018 [6]	Randomized controlled trial	NCT01795495	USA	1 September 2013–1 September 2016	3 days	Adolescents undergoing posterior spinal fusion for idiopathic scoliosis	63	Methadone	0.1 mg/kg IV + remifentanil	22	Remifentanil or remifentanil + magnesium	NR	38
Fons 2024 [5]	Randomized controlled trial	NCT02558010	USA	February 2016–October 2021	3 days	English-speaking adolescents (10–18 years) who underwent a posterior spinal fusion planned for ≥10 levels for idiopathic scoliosis	58	Methadone	0.2 mg/kg IV divided into two doses of 10 mg each	30	Morphine	0.1 mg/kg	28
Uhrbrand 2025 [8]	Randomized controlled trial	NCT04764825	Denmark	26 February 2021 to 19 June 2024	3 months	Patients aged 18–80 undergoing lumbar spine fusion	124	Methadone (pre-incision or pre-closure)	0.2 mg/kg infusion	85	Morphine	0.2 mg/kg	39

**Abbreviations:** IV, intravenous; kg, kilogram; mg, milligram; N, total sample size; n, group sample size; NCT, National Clinical Trial number; NR, not reported; RCT, randomized controlled trial; USA, United States of America; µg, microgram.

**Table 2:** Baseline characteristics of the included study groups.

Study	Arm	Age, Years	Male Sex, n (%)	BMI, kg/m^2^	Weight, kg	Height, cm	ASA I, n (%)	ASA II, n (%)	ASA III, n (%)
Gottschalk 2011 [4]	Methadone	62.9 ± 9.5	5 (38.5%)	26.3 ± 4.6	73.6 ± 16.18	167 ± 11.1	1 (7.7%)	12 (92.3%)	0 (0%)
	Sufentanil	53.1 ± 15	6 (37.5%)	28.1 ± 4.7	79.2 ± 15.6	168 ± 10.1	0 (0%)	16 (100%)	0 (0%)
Murphy 2017 [9]	Methadone	64.5 (57–71)	32 (51.6%)	NR	83.3 ± 20.8	170.2 (160–180.3)	NR	NR	NR
	Hydromorphone	60 (45–66)	21 (39.6%)	NR	80.7 ± 22.1	167.6 (160–172.7)	NR	NR	NR
Martin 2018 [6]	Methadone	15.4 ± 1.2	5 (23%)	22.8 ± 5.7	64.5 ± 20	NR	5 (23%)	17 (77%)	0 (0%)
	Remifentanil	14.2 ± 1.4	3 (16%)	21.6 ± 4.3	55.9 ± 11.8	NR	7 (37%)	12 (63%)	0 (0%)
	Magnesium	15.3 ± 1.9	3 (16%)	24.2 ± 5.6	63.1 ± 15.6	NR	7 (37%)	0 (58%)	1 (5%)
Fons 2024 [5]	Methadone	15 ± 1.8	4 (16%)	NR	58.2 ± 12.8	NR	NR	NR	NR
	Morphine	14.6 ± 2.3	5 (22.7%)	NR	57.5 ± 11.8	NR	NR	NR	NR
Uhrbrand 2025 [8]	Methadone pre-incision	64.9 ± 12.2	16 (41%)	26.7 (23.7–30.1)	NR	NR	3 (8%)	24 (62%)	12 (30%)
	Methadone pre-wound closure	63.7 ± 11.7	12 (32%)	26.6 (23.1–31.2)	NR	NR	7 (18%)	22 (58%)	9 (24%)
	Morphine	63.8 ± 10.8	17 (47%)	28 (24.8–33.8)	NR	NR	6 (17%)	21 (58%)	9 (25%)

Continuous variables are presented as mean ± SD or median (IQR), as reported. Abbreviations: ASA, American Society of Anesthesiologists physical status classification; BMI, body mass index; IQR, interquartile range; n, number of participants; NR, not reported; SD, standard deviation.

## Data Availability

All data are provided in the manuscript and its related Supplementary Material.

## Supplementary Materials

The following supporting information can be downloaded at https://www.mdpi.com/article/10.3390/jcm15218470/s1: Table S1: Search strategy; Table S2: GRADE Certainty of evidence; Figure S1: Leave-one-out sensi-tivity analysis of postoperative pain intensity at 24 h; Figure S2: Leave-one-out sensitivity analysis of postoperative opioid consumption at 24 h; Figure S3: Doi plot for postoperative pain intensity at 24 h; Figure S4: Doi plot for postoperative opioid consumption at 24 h; Supplementary file: PRISMA Checklist.

## Abbreviations

NMDA: N-methyl-D-aspartate; GRADE: Grading of Recommendations Assessment, Development and Evaluation; MD: mean difference; CI: confidence interval; SMD: standardized mean difference; RR: risk ratio; PRISMA: Preferred Reporting Items for Systematic Reviews and Meta-Analyses; PROSPERO: International Prospective Register of Systematic Reviews; I^2^: I-squared statistic; τ^2^: tau-squared statistic; LFK: Luis Furuya-Kanamori; NSAIDs: nonsteroidal anti-inflammatory drugs; COX-2: cyclooxygenase-2; ERAS: Enhanced Recovery After Surgery; MCID: minimal clinically important difference.
