# Supplementary material for: Intraoperative Intravenous Methadone Versus Non-Methadone Opioid Analgesia in Instrumented Spinal Fusion: A GRADE-Guided Systematic Review and Meta-Analysis
Source: J Clin Med. 2026 Oct 30;15(21):8470. doi: 10.3390/jcm15218470 (PMC13763907; doi:10.3390/jcm15218470)
Supplement: Supplementary file 1 [file jcm-15-08470-s001.zip › jcm-4599989-supplementary.pdf]

Supplementary Materials

Table S1. Search strategy.

Table S2. GRADE Certainty of evidence.

Figure S1: Leave-one-out sensitivity analysis of postoperative pain intensity at 24 hours.

Figure S2: Leave-one-out sensitivity analysis of postoperative opioid consumption at 24 hours.

Figure S3: Doi plot for postoperative pain intensity at 24 hours.

Figure S4: Doi plot for postoperative opioid consumption at 24 hours.

PRISMA Checklist.

Database	Search Strategy	N	Search date	Limitations
PubMed	(methadone OR Phenadone OR Physeptone OR Phymet OR Dolophine OR Amidone OR Biodone OR Metadol OR Metasedin OR Methaddict OR Methadose OR Methex OR Pinadone OR Symoron OR "Methadone"[Mesh])<br>AND<br>("spinal fusion*" OR "posterior spinal fusion*" OR "thoracolumbar spinal fusion*" OR "lumbar spinal fusion*" OR Spondylodesis OR Spondylodeses OR Spondylosyndesis OR Spondylosyndeses OR "Spinal Fusion"[Mesh])	28	27 August 2026	No limitations applied
Scopus	(methadone OR Phenadone OR Physeptone OR Phymet OR Dolophine OR Amidone OR Biodone OR Metadol OR Metasedin OR Methaddict OR Methadose OR Methex OR Pinadone OR Symoron)<br>AND<br>("spinal fusion*" OR "posterior spinal fusion*" OR "thoracolumbar spinal fusion*" OR "lumbar spinal fusion*" OR Spondylodesis OR Spondylodeses OR Spondylosyndesis OR Spondylosyndeses)	57	27 August 2026	Title, Abstract, keywords
Web of Science	(methadone OR Phenadone OR Physeptone OR Phymet OR Dolophine OR Amidone OR Biodone OR Metadol OR Metasedin OR Methaddict OR Methadose OR Methex OR Pinadone OR Symoron)<br>AND<br>("spinal fusion*" OR "posterior spinal fusion*" OR "thoracolumbar spinal fusion*" OR "lumbar spinal fusion*" OR Spondylodesis OR Spondylodeses OR Spondylosyndesis OR Spondylosyndeses)	34	27 August 2026	Topic
Cochrane Library	(methadone OR Phenadone OR Physeptone OR Phymet OR Dolophine OR Amidone OR Biodone OR Metadol OR Metasedin OR Methaddict OR Methadose OR Methex OR Pinadone OR Symoron)<br>AND<br>("spinal fusion*" OR "posterior spinal fusion*" OR "thoracolumbar spinal fusion*" OR "lumbar spinal fusion*" OR Spondylodesis OR Spondylodeses OR Spondylosyndesis OR Spondylosyndeses)	20	27 August 2026	Title, Abstract, keywords
Number of studies before removal of duplicates		139		
Number of studies after removal of duplicates		91		

Supplementary Table S1. Search strategy.

Certainty assessment							Summary of findings				
Participants (studies) Follow-up	Risk of bias	Inconsistency	Indirectness	Imprecision	Publication bias	Overall certainty of evidence	Study event rates (%)		Relative effect (95% CI)	Anticipated absolute effects	
							With control	With methadone		Risk with control	Risk difference with methadone
**Postoperative pain at 24 hours (0–10 pain scale)**											
185 (3 RCTs)	serious[a]	not serious	not serious	serious[b]	not suspected[h]	⊕⊕◯◯ Low	88	97	—	—	MD **1.06 points lower** (2.03 lower to 0.09 lower)
**Postoperative pain at 48 hours (0–10 pain scale)**											
144 (2 RCTs)	serious[a]	not serious	not serious	serious[b]	not suspected[h]	⊕⊕◯◯ Low	69	75	—	—	MD **1.52 points lower** (2.36 lower to 0.67 lower)
**Postoperative pain at 72 hours (0–10 pain scale)**											
144 (2 RCTs)	serious[a]	not serious	not serious	serious[b]	not suspected[h]	⊕⊕◯◯ Low	69	75	—	—	MD **1.04 points lower** (1.91 lower to 0.17 lower)
**Postoperative opioid consumption at 24 hours**											
192 (4 RCTs)	serious[a]	not serious	not serious	not serious	not suspected[h]	⊕⊕⊕◯ Moderate	93	99	—	—	SMD **0.50 SD lower** (0.79 lower to 0.21 lower)

Postoperative opioid consumption at 48 hours

76<br>(2 RCTs)	serious[a]	serious[c]	not serious	serious[d]	not suspected[h]	⊕◯◯◯<br>Very low	38	38	—	—	SMD **0.54 SD lower**<br>(1.72 lower to 0.63 higher)

Vomiting (follow-up: up to 72 hours)

76<br>(2 RCTs)	serious[a]	not serious	not serious	serious[e]	not suspected[h]	⊕⊕◯◯<br>Low	16/38<br>(42.1%)	18/38<br>(47.4%)	**RR 1.10**<br>(0.72 to 1.70)	421 per 1,000	**42 more per 1,000**<br>(118 fewer to 295 more)

Respiratory events (follow-up: PACU to 72 hours)

104<br>(2 RCTs)	serious[a]	not serious	serious[f]	very serious[g]	not suspected[h]	⊕◯◯◯<br>Very low	6/52<br>(11.5%)	2/52<br>(3.8%)	**RR, 0.18**<br>(0.04 to 0.69)	115 per 1,000	**94 fewer per 1,000**<br>(110 fewer to 36 fewer)

Supplementary Table S2. GRADE Certainty of evidence.

Abbreviations: CI: confidence interval; **IV:** intravenous; **MD:** mean difference; **PACU:** post-anesthesia care unit; **RCT:** randomized controlled trial; **RR:** risk ratio; **SMD:** standardized mean difference

Explanations:

a. Downgraded one level for risk of bias because at least half of the contributing trials had some concerns in one or more RoB 2 domains; no included trial was judged at high risk.

b. Downgraded one level for imprecision: the confidence interval includes an effect smaller than a 1-point minimally important difference on a 0–10 pain scale, and the estimate is based on only two or three trials.

c. Downgraded one level for inconsistency because heterogeneity was substantial ($I^2 = 82.6\%$) and study estimates differed materially.

d. Downgraded one level for imprecision because the confidence interval includes appreciable benefit, no effect, and possible harm in a small sample.

e. Downgraded one level for imprecision because the confidence interval includes both appreciable benefit and appreciable harm.

f. Downgraded one level for indirectness because respiratory outcomes used different definitions and observation windows (PACU hypoventilation versus broader postoperative respiratory depression).

g. Downgraded two levels for imprecision because only eight events occurred and the confidence interval includes substantial benefit, no effect, and possible harm.

h. Formal assessment of publication bias was not reliable because fewer than 10 studies contributed to every outcome; no downgrade was applied.

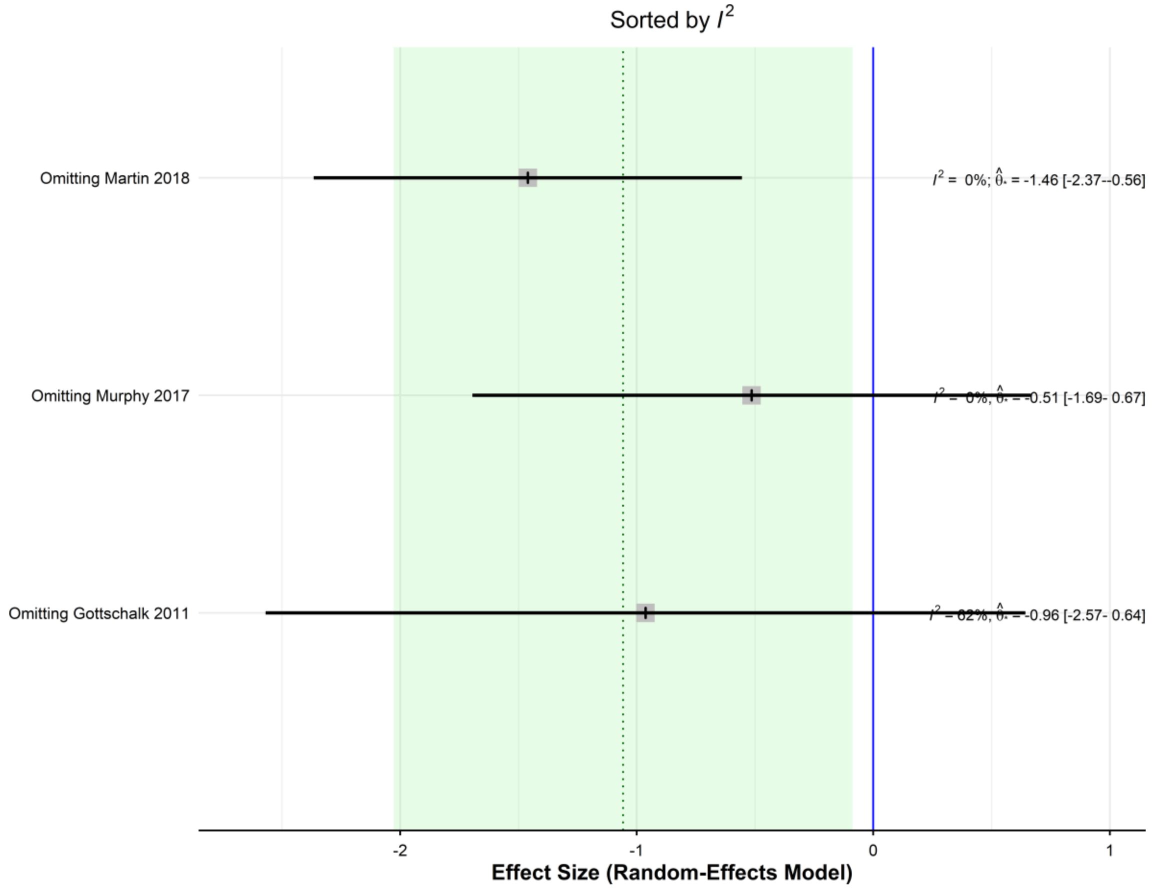

Figure S1. Leave-one-out sensitivity analysis of postoperative pain intensity at 24 hours. [4,6,9]

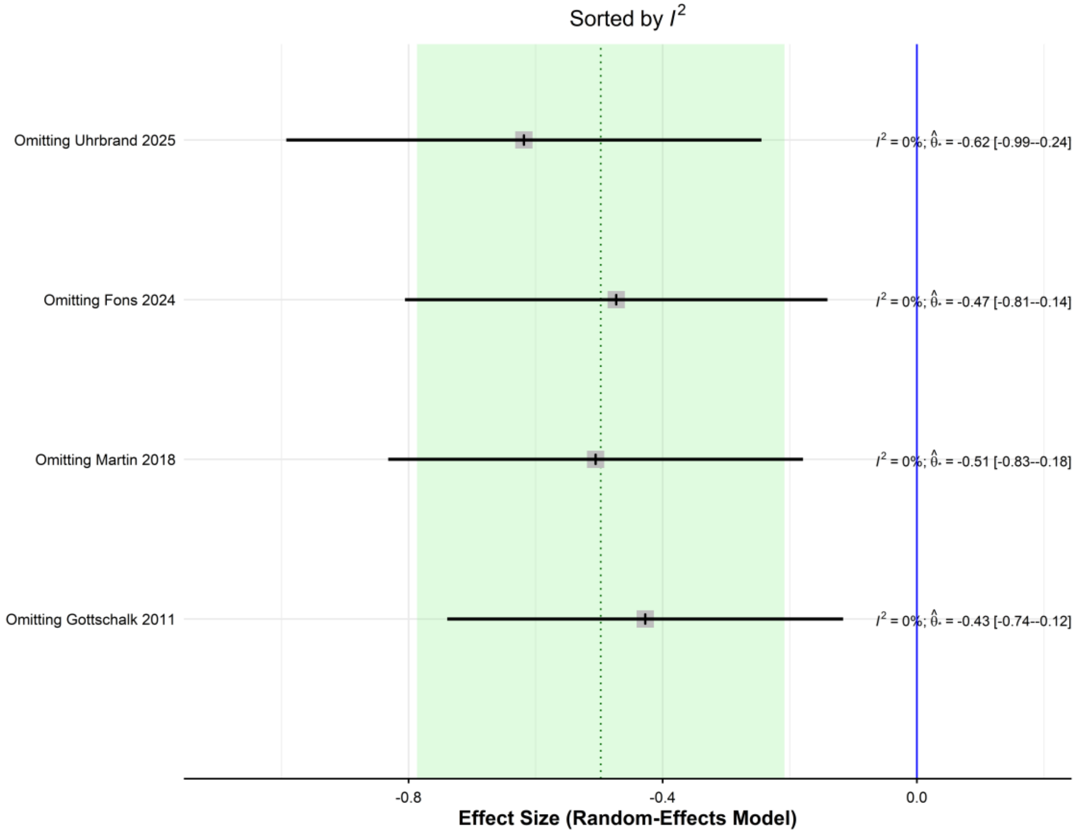

Figure S2. Leave-one-out sensitivity analysis of postoperative opioid consumption at 24 hours. [4,5,6,8]

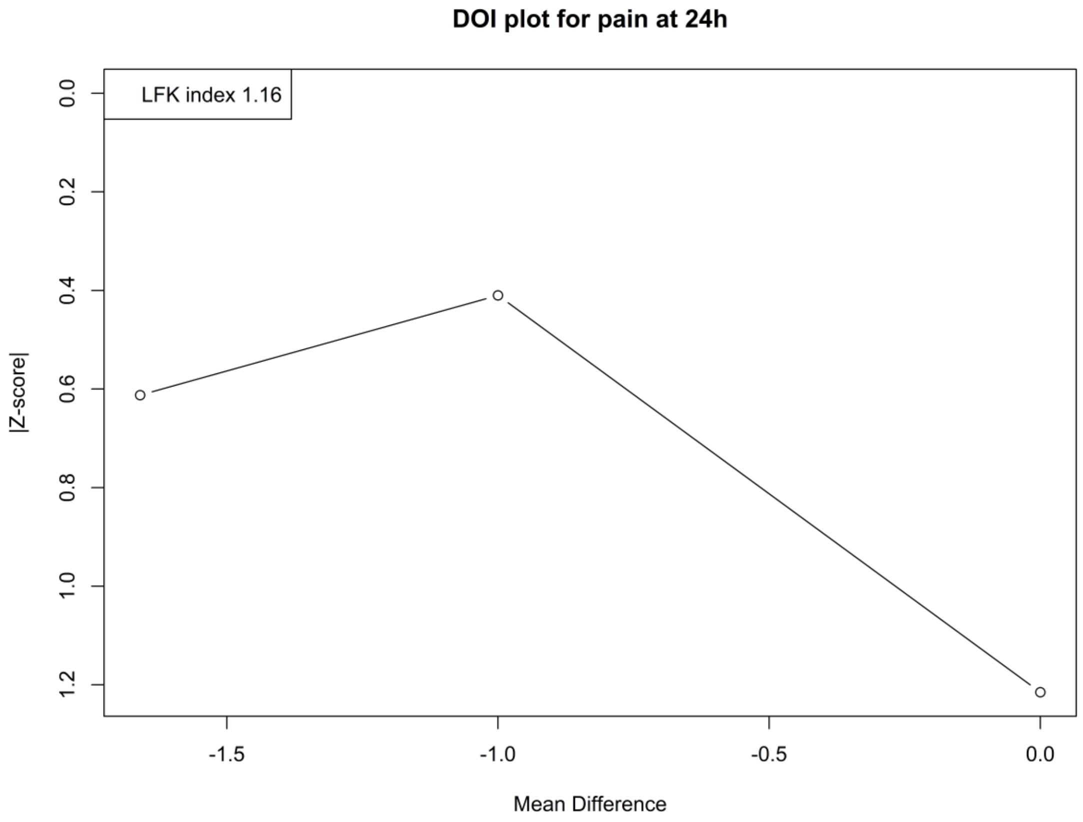

Figure S3. Doi plot for postoperative pain intensity at 24 hours (LFK index, 1.16).

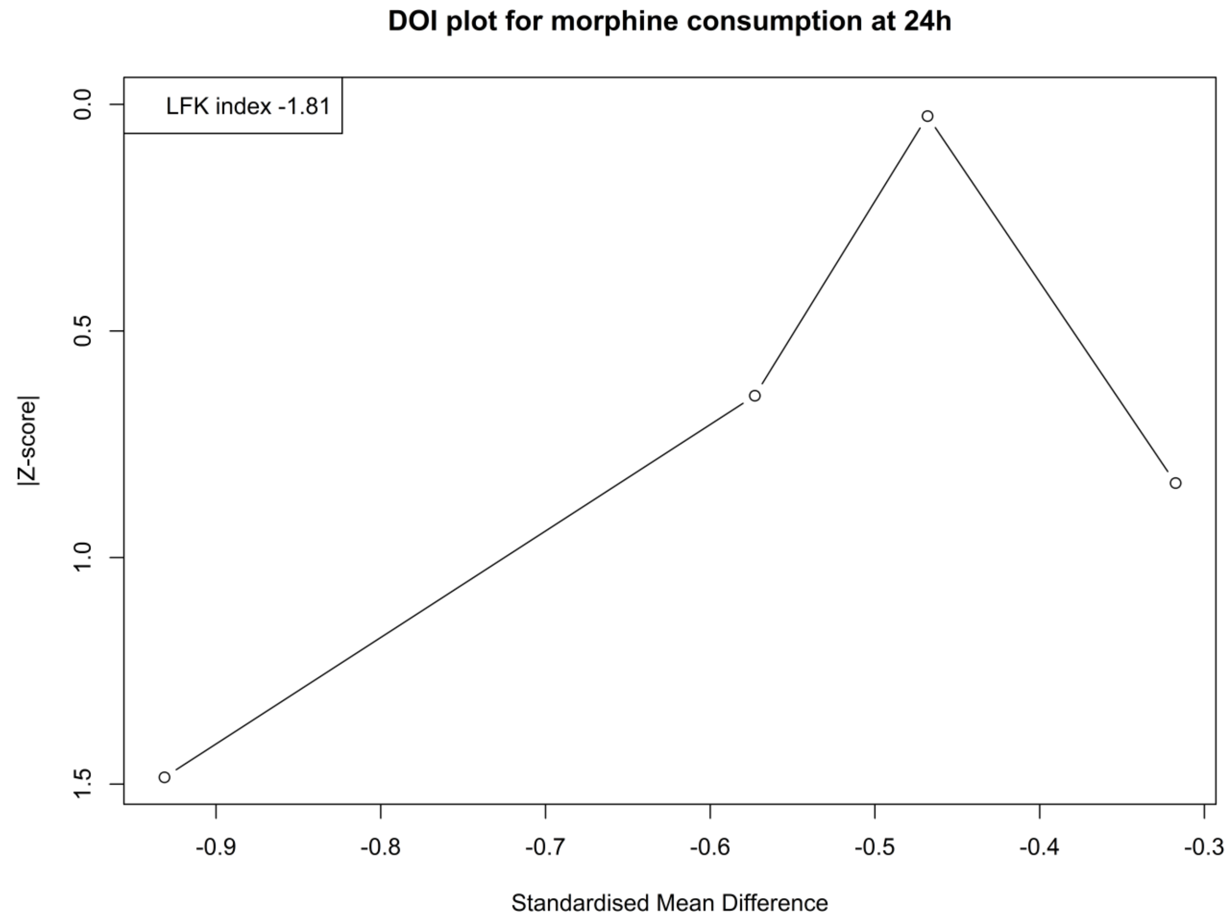

Figure S4. Doi plot for postoperative opioid consumption at 24 hours (LFK index, -1.81).

Section and Topic	Item #	Checklist item	Location where item is reported
**TITLE**			
Title	1	Identify the report as a systematic review.	1
**ABSTRACT**			
Abstract	2	See the PRISMA 2020 for Abstracts checklist.	1
**INTRODUCTION**			
Rationale	3	Describe the rationale for the review in the context of existing knowledge.	2
Objectives	4	Provide an explicit statement of the objective(s) or question(s) the review addresses.	2-3
**METHODS**			
Eligibility criteria	5	Specify the inclusion and exclusion criteria for the review and how studies were grouped for the syntheses.	3
Information sources	6	Specify all databases, registers, websites, organisations, reference lists and other sources searched or consulted to identify studies. Specify the date when each source was last searched or consulted.	3
Search strategy	7	Present the full search strategies for all databases, registers and websites, including any filters and limits used.	3
Selection process	8	Specify the methods used to decide whether a study met the inclusion criteria of the review, including how many reviewers screened each record and each report retrieved, whether they worked independently, and if applicable, details of automation tools used in the process.	5
Data collection process	9	Specify the methods used to collect data from reports, including how many reviewers collected data from each report, whether they worked independently, any processes for obtaining or confirming data from study investigators, and if applicable, details of automation tools used in the process.	5
Data items	10a	List and define all outcomes for which data were sought. Specify whether all results that were compatible with each outcome domain in each study were sought (e.g. for all measures, time points, analyses), and if not, the methods used to decide which results to collect.	5
	10b	List and define all other variables for which data were sought (e.g. participant and intervention characteristics, funding sources). Describe any assumptions made about any missing or unclear information.	5
Study risk of bias assessment	11	Specify the methods used to assess risk of bias in the included studies, including details of the tool(s) used, how many reviewers assessed each study and whether they worked independently, and if applicable, details of automation tools used in the process.	5
Effect measures	12	Specify for each outcome the effect measure(s) (e.g. risk ratio, mean difference) used in the synthesis or presentation of results.	5-6
Synthesis methods	13a	Describe the processes used to decide which studies were eligible for each synthesis (e.g. tabulating the study intervention characteristics and comparing against the planned groups for each synthesis (item #5)).	5-6
	13b	Describe any methods required to prepare the data for presentation or synthesis, such as handling of missing summary statistics, or data conversions.	5-6
	13c	Describe any methods used to tabulate or visually display results of individual studies and syntheses.	5-6
	13d	Describe any methods used to synthesize results and provide a rationale for the choice(s). If meta-analysis was performed, describe the model(s), method(s) to identify the presence and extent of statistical heterogeneity, and software package(s) used.	5-6
	13e	Describe any methods used to explore possible causes of heterogeneity among study results (e.g. subgroup analysis, meta-regression).	5-6
	13f	Describe any sensitivity analyses conducted to assess robustness of the synthesized results.	5-6
Reporting bias assessment	14	Describe any methods used to assess risk of bias due to missing results in a synthesis (arising from reporting biases).	5
Certainty assessment	15	Describe any methods used to assess certainty (or confidence) in the body of evidence for an outcome.	6
**RESULTS**			

Section and Topic	Item #	Checklist item	Location where item is reported
Study selection	16a	Describe the results of the search and selection process, from the number of records identified in the search to the number of studies included in the review, ideally using a flow diagram.	6-7
	16b	Cite studies that might appear to meet the inclusion criteria, but which were excluded, and explain why they were excluded.	6-7
Study characteristics	17	Cite each included study and present its characteristics.	7-8
Risk of bias in studies	18	Present assessments of risk of bias for each included study.	8
Results of individual studies	19	For all outcomes, present, for each study: (a) summary statistics for each group (where appropriate) and (b) an effect estimate and its precision (e.g. confidence/credible interval), ideally using structured tables or plots.	9-12
Results of syntheses	20a	For each synthesis, briefly summarise the characteristics and risk of bias among contributing studies.	9-12
	20b	Present results of all statistical syntheses conducted. If meta-analysis was done, present for each the summary estimate and its precision (e.g. confidence/credible interval) and measures of statistical heterogeneity. If comparing groups, describe the direction of the effect.	9-12
	20c	Present results of all investigations of possible causes of heterogeneity among study results.	9-12
	20d	Present results of all sensitivity analyses conducted to assess the robustness of the synthesized results.	9-12
Reporting biases	21	Present assessments of risk of bias due to missing results (arising from reporting biases) for each synthesis assessed.	8
Certainty of evidence	22	Present assessments of certainty (or confidence) in the body of evidence for each outcome assessed.	12
**DISCUSSION**			
Discussion	23a	Provide a general interpretation of the results in the context of other evidence.	12-14
	23b	Discuss any limitations of the evidence included in the review.	14-15
	23c	Discuss any limitations of the review processes used.	14-15
	23d	Discuss implications of the results for practice, policy, and future research.	14, 15
**OTHER INFORMATION**			
Registration and protocol	24a	Provide registration information for the review, including register name and registration number, or state that the review was not registered.	3
	24b	Indicate where the review protocol can be accessed, or state that a protocol was not prepared.	3
	24c	Describe and explain any amendments to information provided at registration or in the protocol.	3
Support	25	Describe sources of financial or non-financial support for the review, and the role of the funders or sponsors in the review.	16
Competing interests	26	Declare any competing interests of review authors.	16
Availability of data, code and other materials	27	Report which of the following are publicly available and where they can be found: template data collection forms; data extracted from included studies; data used for all analyses; analytic code; any other materials used in the review.	16

PRISMA Checklist
